# Recent trends in the nanozeolites-based oxygen concentrators and their application in respiratory disorders

**DOI:** 10.3389/fmed.2023.1147373

**Published:** 2023-04-27

**Authors:** Virendra Kumar Yadav, Nisha Choudhary, Gajendra Kumar Inwati, Ashita Rai, Bijendra Singh, Bharat Solanki, Biswaranjan Paital, Dipak Kumar Sahoo

**Affiliations:** ^1^Department of Biosciences, School of Liberal Arts and Sciences, Mody University of Science and Technology, Lakshmangarh, Rajasthan, India; ^2^Department of Life Sciences, Hemchandracharya North Gujarat University, Patan, Gujarat, India; ^3^Department of Environment Sciences, School of Sciences, P P Savani University, Surat, Gujarat, India; ^4^Department of Chemistry, Medicaps University, Indore, Madhya Pradesh, India; ^5^School of Environment and Sustainable Development, Central University of Gujarat, Gandhinagar, Gujarat, India; ^6^School of Chemical Sciences, Central University of Gujarat, Gandhinagar, Gujarat, India; ^7^Department of Biochemistry, M B Patel Science College, Anand, Gujarat, India; ^8^Redox Regulation Laboratory, Department of Zoology, College of Basic Science and Humanities, Odisha University of Agriculture and Technology, Bhubaneswar, India; ^9^Department of Veterinary Clinical Sciences, College of Veterinary Medicine, Iowa State University, Ames, IA, United States

**Keywords:** chabazite, microbial infection, molecular sieves, nano-zeolite, oxygen therapy, pneumonia, respiratory disorders

## Abstract

Medical-grade oxygen is the basic need for all medical complications, especially in respiratory-based discomforts. There was a drastic increase in the demand for medical-grade oxygen during the current pandemic. The non-availability of medical-grade oxygen led to several complications, including death. The oxygen concentrator was only the last hope for the patient during COVID-19 pandemic around the globe. The demands also are everlasting during other microbial respiratory infections. The yield of oxygen using conventional molecular zeolites in the traditional oxygen concentrator process is less than the yield noticed when its nano-form is used. Nanotechnology has enlightened hope for the efficient production of oxygen by such oxygen concentrators. Here in the current review work, the authors have highlighted the basic structural features of oxygen concentrators along with the current working principle. Besides, it has been tried to bridge the gap between conventional oxygen concentrators and advanced ones by using nanotechnology. Nanoparticles being usually within 100 nm in size have a high surface area to volume ratio, which makes them suitable adsorbents for oxygen. Here authors have suggested the use of nano zeolite in place of molecular zeolites in the oxygen concentrator for efficient delivery of oxygen by the oxygen concentrators.

## 1. Introduction

Oxygen is the basic requirement for both a healthy and a diseased individual. There are certain medical diseases, especially cardiovascular respiratory disorders, where there is a requirement for pure or concentrated oxygen by the patient (1, 2). This pure or concentrated oxygen is indeed known as medical oxygen, and in the absence of it, a patient may experience hypoxemia [low levels of oxygenated blood (3)]. Hypoxemia is caused by a range of common conditions—including childhood pneumonia, newborn conditions (4, 5), and obstetric emergencies (6, 7). The body of the sufferer will experience severe detrimental consequences from hypoxemia on the cells that carry out crucial biological functions (8). The atmospheric air with a 21% O_2_ concentration and when its percentage is below 19.5% by volume and/or highly elevated CO_2_ level in air is called an oxygen-deficient condition (9). The O_2_ deficient in body a comparatively O_2_ less environment can lead to a condition called asphyxiation (10). When severe hypoxemia is not quickly diagnosed and addressed, it can lead to death.

For a healthy individual, 21% of oxygen is sufficient, but for a patient suffering from a respiratory disease or cardiovascular then, there is a requirement for concentrated or pure oxygen or medical grade oxygen. Oxygen is listed as an essential drug in India. If pure oxygen is not provided to the patient on time, during hypoxemia, then the lung will be devoid of oxygenated blood, from where the oxygen will not be supplied to the vital organs of the body (11). As a result, it will lead to damage of organs and, ultimately, the death of the patients. Based on the application, oxygen can be classified into two classes: medical and industrial grade. Medical grade oxygen is highly pure (12) in order to supply concentrated oxygen to the patient, while impurities could be permitted in the industrial grade, which is needed in steel making, etc. Moreover, medical-grade oxygen should be supplied in sterilized cylinders in order to prevent other diseases as per the gas cylinder rule 1981 (13, 14).

For the patient’s recovery from several illnesses, including respiratory infections such as asthma, emphysema, and perhaps other severe acute respiratory syndrome (SARS)-coronavirus-2 (CoV2) infestations, supplemental oxygen is recommended (15). Oxygen therapy is provided by means of an oxygen cylinder, oxygen generation plant, and oxygen concentrators. The demand for oxygen concentrators has grown drastically due to the SARS-CoV_2_. In order to help patients with lower blood oxygen levels, oxygen concentrators are biomedical equipment that typically concentrates oxygen from the atmosphere (16). Oxygen concentrators have been used for the last several decades, especially in low-income countries, where the medical facility is poorly developed, or in remote or hilly areas where hospitals are very far. Oxygen concentrators could be either fixable or static, or they could be mobile or portable (16).

As the word “*concentrator*” indicates the concentration or enrichment of anything, and here oxygen is being concentrated from the air mixture. So, in layman’s language, we can say that this device concentrates oxygen from the air by eliminating nitrogen from them. Our atmospheric air contains a mixture of gases, mainly nitrogen (78%), oxygen (21%), and a small number of other gases. So, the main purpose of this device is to eliminate the nitrogen (which is present in a larger amount) from the air and supply the residual component, i.e., oxygen up to 90% purity, to the patient. When the air is sent through an oxygen concentrator, it is converted to 90–95 percent pure oxygen and 5–10 percent nitrogen. Since it is challenging to obtain that amount of oxygen alone without the aid of medical equipment, the nitrogen is segregated to ensure that patients receive the maximum dose of oxygen available. Home-based oxygen concentrators have numerous advantages compared to oxygen cylinders, which may leak or burst (17). Compared to conventional oxygen cylinders, oxygen concentrators are significantly less hazardous (18). Old-fashioned oxygen cylinders have been replaced with household and transportable oxygen concentrators that can “generate” their own oxygen (19). From time to time, there were several modifications also in the oxygen concentrator; for instance, few investigators used it alone, few used it with other oxygen devices like ventilators (20), and few used nanomaterials in place of micron-sized zeolites to increase the efficiency of the concentrator (21). Molecular zeolites are artificially produced molecular sieves. They are prepared from alkali metal aluminosilicates. They have common applications in the gas chromatography also to sieve the molecules. The calcium aluminosilicate has pore diameter of 0.5 nm, while sodium aluminosilicate has pore diameter of 1 nm. They are produced using nanotechnology tools and techniques. According to some investigators, especially from Africa, Nigeria, where continuous electricity supply is a big challenge, the oxygen concentrators were fitted with solar panels for a continuous, uninterrupted supply of oxygen to the patient (18, 22).

**Figure 1:**
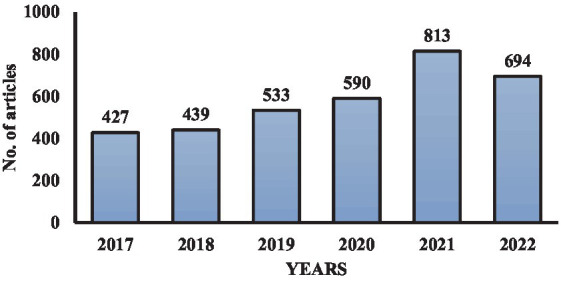
Year-wise articles published in the scientific domain on oxygen concentrators.

We searched oxygen concentrators and portable oxygen concentrators on Science Direct for the time frame of 2017–2022, and the authors found a sharp increase in the number of articles in these 6 years. The key word used for this search was “oxygen concentrators.” The number of articles drastically increased after the COVID-19 pandemic. Figure 1 shows the articles available in Science Direct within this time frame. So, from this research, we tried to bridge this gap with the latest information on oxygen concentrators which may help medical professionals in the future. It may also help in increasing the efficiency of president oxygen concentrators by using advancements in technology.

In the current review work, emphasis is given to the basic working principle of oxygen concentrators. Besides this, recent trends in the utilization of portable oxygen concentrators have been discussed. Nanosized zeolites are produced from the molecular zeolites using nanotechnology tools. These are crystalline microporous solids and have physicochemical properties similar to those of micron-sized crystals. The method for increasing the efficiency of current oxygen concentrators by using nanosized molecular zeolites is elegantly reviewed with an objective that the use of nanosized molecular zeolites may solve the demands of oxygen supply in a future pandemic or any disease outbreak.

## 2. Types of oxygen based on their applications

There are essentially two sorts of oxygen depending upon grade: medical and industrial. Many industries run their production facilities using commercial oxygen (23). Manufacturing oxygen for industrial purposes is also required to meet industrial standards. Oxygen plants used in the generation of oxygen for industrial applications must meet the specifications of the industry. The procedure for generating medical and industrial oxygen is the same. Furthermore, medical oxygen is produced with a significant purity level, and the equipment used to produce it is made to the best of its ability (24). Medical oxygen is ultra-pure oxygen that has been created for use in the human body and is utilized in medical procedures. Medical oxygen is purified in the air separation process until it fulfills regulatory requirements (25).

**Figure 2:**
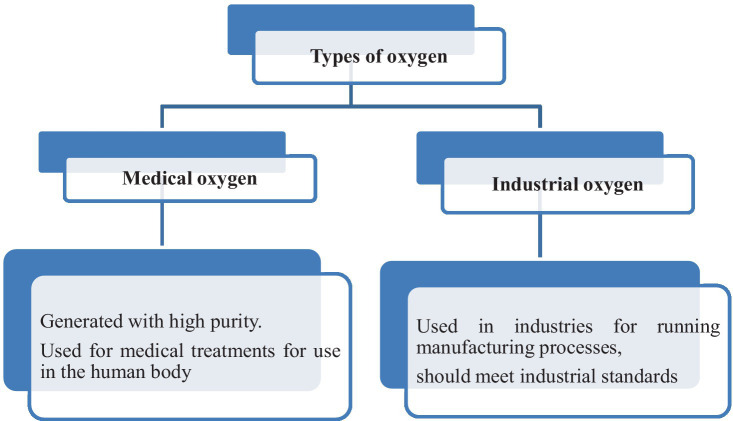
Medical oxygen versus industrial oxygen.

In operating rooms, emergency departments, and ambulances, medical-grade oxygen is being used. Medical oxygen production requires adequate drug licenses and adherence to standard operating procedures, and it is recognized as a drug on the World Health Organization (WHO)’s list of essential medicines. To generate medical oxygen, it is also required to adhere to Indian Pharmacopeia (IP) guidelines (25). Figure 2 shows the types of oxygen as per the requirement and applications.

### 2.1. Liquid medical oxygen

Highly pure oxygen in the form of liquid medical oxygen (LMO) is being used for medical care and was invented for use in the human body (26). Liquification promotes smoother conveyance plus greater storage. These kinds of LMO can indeed be kept in a mobile cylinder that is attached to the main conduit. Due to the high concentration of liquid oxygen, a compact reservoir may hold much of the gas. Sarkar et al. in 2014 described the importance of liquid ventilation in treating several diseases and considered it effective over oxygen in the form of gas (4, 27).

## 3. Oxygen therapy

Oxygen therapy is the most commonly reliable method to treat hypoxemia in cardiovascular and respiratory system-related disorders (28, 29) and during pandemic disease outbreaks. All these patients require a continuous supply of medical-grade oxygen for the proper functioning of their vital organs and body. The method of distribution and equipment used for oxygen therapy influence the selection of variables, such as the patient’s individual requirements and the discretion of the treating hospital personnel (30). Oxygen cylinders, oxygen production facilities, and oxygen concentrators are used to administer oxygen therapy.

### 3.1. Areas where oxygen therapy is required

**Figure 3:**
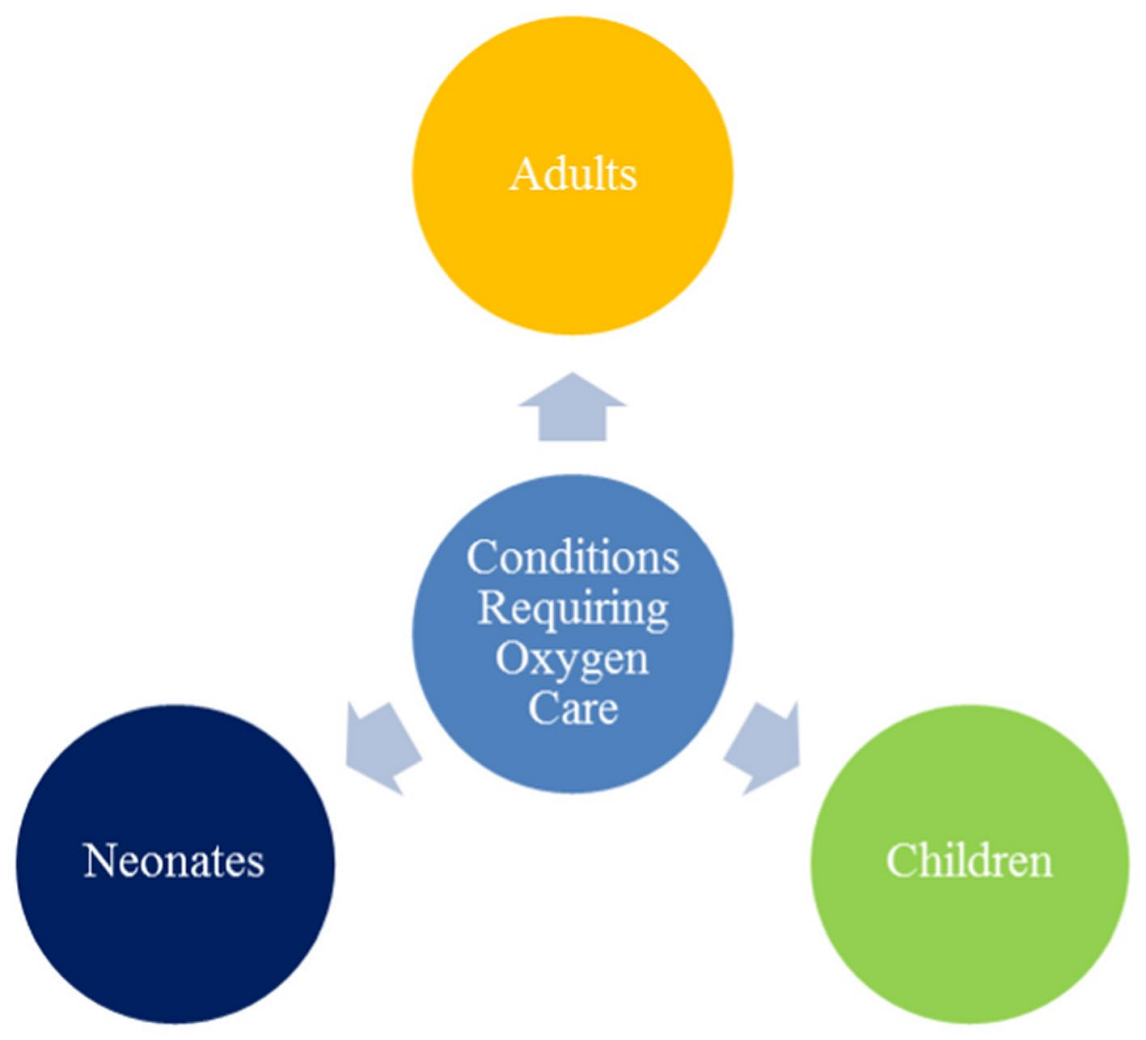
Conditions requiring oxygen care in clinical cases.

Multiple chronic and acute medical disorders, including chronic asthma, cystic fibrosis, pulmonary hypertension, obstructive sleep apnea, heart failure, cases of anaphylaxis, severe trauma, seizure, or hypothermia, are managed by oxygen therapy (31–33). Whenever hypoxemia is found in any of the following situations, oxygen therapy is necessary for operating rooms as well. In the case of children and neonates, oxygen therapy is required during prematurity, birth asphyxia, acute sepsis, shock, severe pneumonia, meningitis, brain injury, coma, anemia, severe malaria, heart failure, acute asthma, etc. Among adults, oxygen therapy is required during, pneumonia, interstitial lung disease, pulmonary sarcoidosis, lower and upper respiratory infections, pneumoconiosis, severe malaria, meningitis, ischemia, etc. Figure 3 shows various conditions requiring oxygen in different age groups.

#### 3.1.1. In adults

- The main conditions requiring oxygen are hypoxemia, breathlessness, and dyspnea (34).
- Every patient’s regular vitals must include pulse oximetry.
- Any patient with a SpO_2_ below 90% on a pulse oximeter or clinical hypoxemia symptoms should be given oxygen, according to the recommendation (35).
- Under some serious circumstances, such as brain injury/infection/coma, shock, sepsis, severe anemia, heart failure, or severe heart failure.
- Whenever the Oxygen saturation, i.e., SpO_2_ is <94%, oxygen should also be administered (26).
- While seeking medical examination as well as background, every seriously sick person should be given oxygen by paramedics, nurses, and other medical practitioners.

According to the clinical recommendations for oxygen therapy, oxygen should be administered to all patients who are in need.

#### 3.1.2. Oxygen care in neonates

Newborn infants receiving oxygen therapy have lower normal oxygen saturation levels in their body as compared to the older neonates. And the condition of severity in low oxygen concentration in their body of neonates is high if they are delivered prematurely. Walsh et al. in 2009, reported to use oxygen for the treatment of several diseases like respiratory disorders and hypoxia, and the treatment process has positive and detrimental effects on the patients. The investigators reported that the extent of use of oxygen in neonates is not well known but they have shown that such treatment system has a positive role in the arsenal for neonates (36).

Dennery in 2010 reported that using medical oxygen in preterm infants is not recommended as it may have a detrimental effect in the long term in neonates. So, the liberal use of oxygen (blood oxygen saturation of >94%) and restrictive use (resulting in a blood oxygen saturation of <80–85%) are detrimental and may have a long-term negative effect. So, appropriate concentration and duration of oxygen may be effective in neonates and infants (37). Ramji et al. in 2015 reported that the use of oxygen had been challenged earlier in both clinical and biochemical results in neonates due to their adverse outcomes. But now, its use is recommended to treat ill neonates in the delivery room and neonatal intensive care unit (NICU). The use of oxygen has shown improvement in neonate survival (38).

#### 3.1.3. Oxygen care in children’s care

Among the most common respiratory ailments in children under the age of five is pneumonia, which accounts for over 15% of all annual fatalities worldwide. Out of this 15%, almost 13% of children with pneumonia develop hypoxemia (39–41). Pneumonia can be caused by bacteria, fungi and viruses (42). In such cases, the oxygen-holding capacity of alveoli and, ultimately, the lungs get reduced, and child needs urgent pure or medical oxygen. If pulse oximetry and oxygen therapy are consistently accessible, the annual child mortality rate could be reduced. Children living at or above 2,500 m above sea level with <90% blood oxygen levels as measured by pulse oximetry are required to receive oxygen therapy (43, 44). If normal oxygen saturation in children exposed to higher altitudes (>2,500 m above sea level) is lesser than that in children living at sea level, a SpO_2_ level of 87 percent can be considered as a criterion for administering oxygen. Any children with a SpO_2_ below 90% should indeed be administered oxygen while being evaluated by pulse oximetry (45).

## 4. Common oxygen supply system

**Figure 4:**
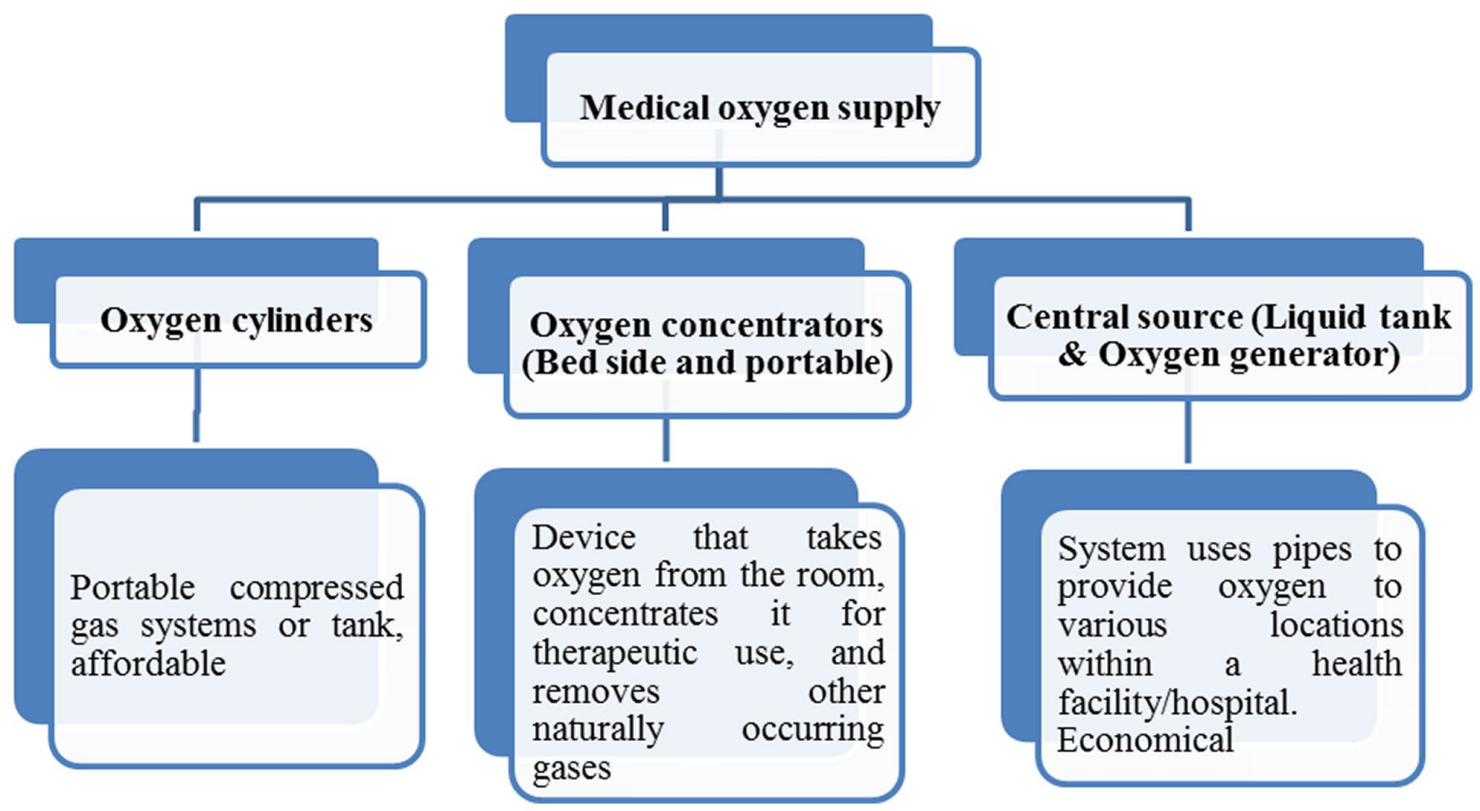
Medical oxygen supply systems.

There are several medical oxygen supply methods to patients, but the most common methods are the form of oxygen cylinders, oxygen concentrators, and liquid tanks & oxygen generators (46). The following methods are mainly used to administer medical oxygen (shown in Figure 4).

## 5. Requirements of medical gas (oxygen) and air points

All critical care areas of the hospitals, such as emergency wards, operation theatres (OT), intensive care unit (ICU), NICU, and labor room, should have an uninterrupted 24 × 7 h oxygen supply. Besides this high dependency unit (HDU) should also have a proper oxygen supply. As per industry norms, the requirement of oxygen supply in hospitals should be as shown in Table 1.

**Table 1:** Requirements of oxygen supply in hospitals as per industry norms.

Specialties/wards	Number of outlet points per bed		
Specialties/wards	Number of outlets points		
	Oxygen	Suction	Air
Intensive care unit	2	2	1
Pediatric intensive care unit/neonatal intensive care unit	3	3	3
High dependency unit	2	2	1
Wards	1	1	1
Operation theater	2	2	1
Labor room	2	2	1
Cardiac intensive care units	2	2	1

ICU, intensive care units; PICU, pediatric intensive care unit; NICU, neonatal intensive care unit.

## 6. Side effects of breathing pure oxygen

**Figure 5:**
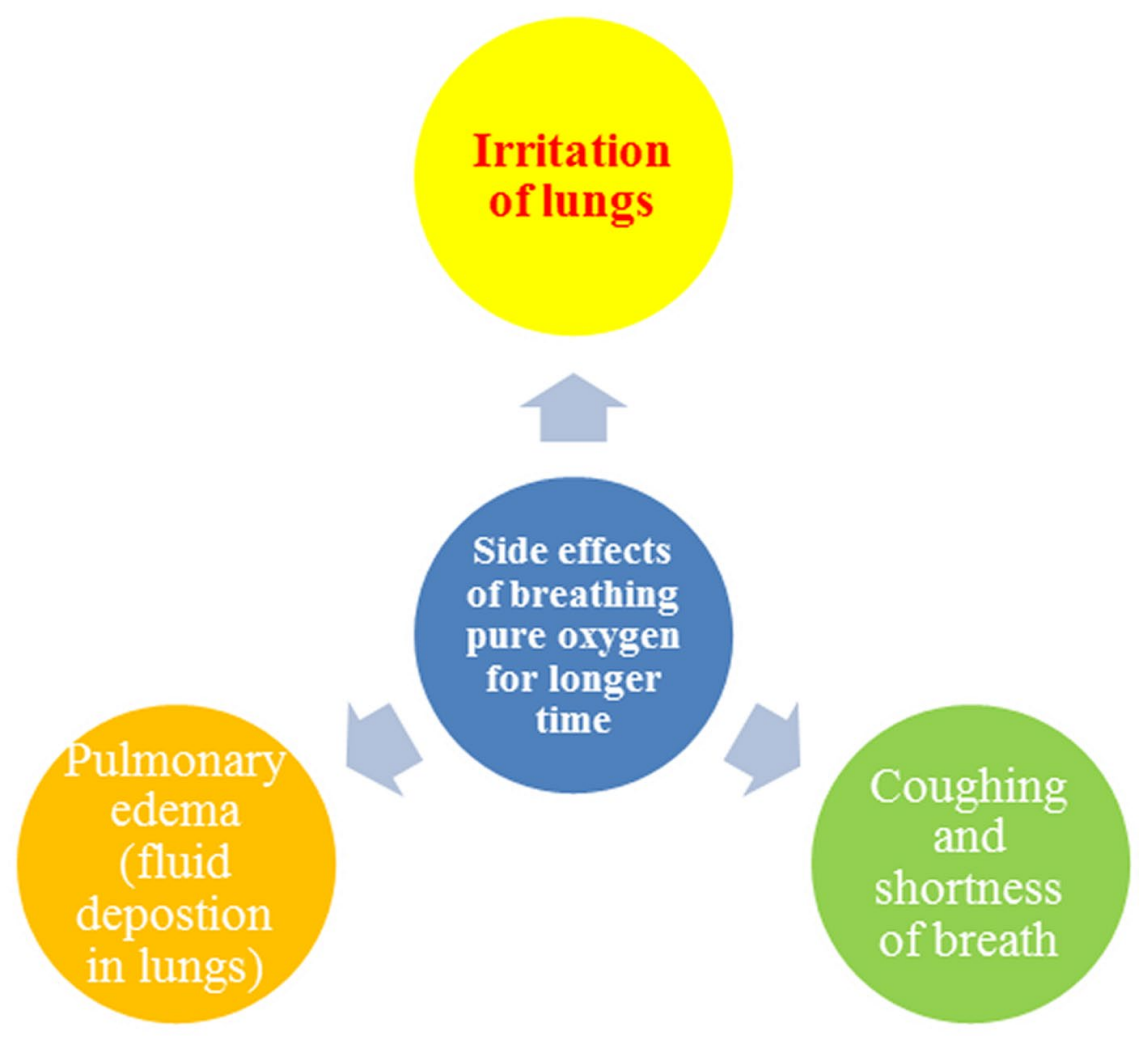
Side effects of breathing pure oxygen.

**Figure 6:**
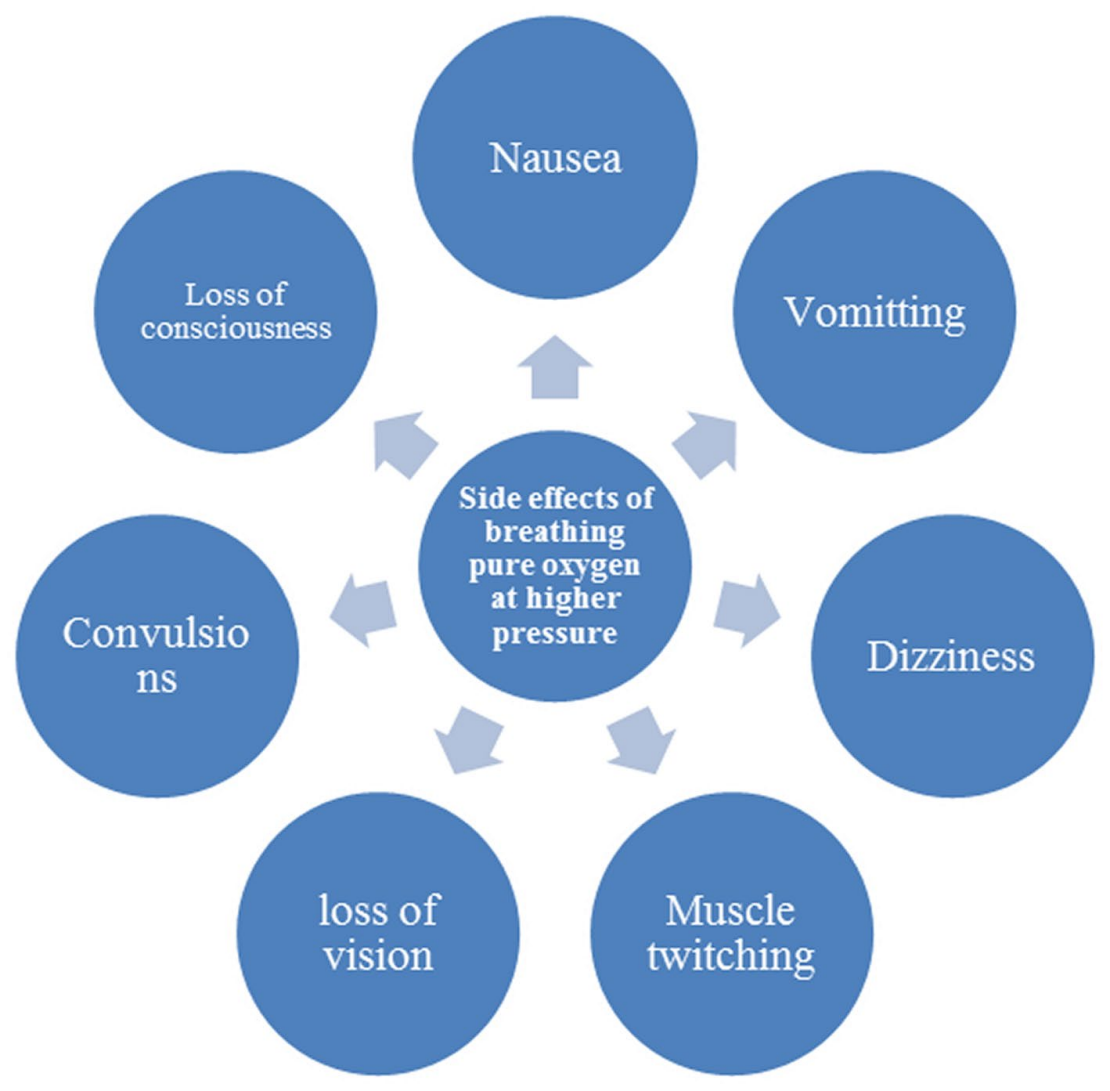
Side effects of breathing pure oxygen at high pressure.

Even though a low level of oxygen in the blood is not ideal, this does not entail that breathing extremely pure oxygen at high pressure and for long periods is beneficial to a patient’s health. Breathing oxygen for a longer time and breathing oxygen at high pressure negatively impact an individual’s health. Breathing pure oxygen for a longer time could lead to the following symptoms and diseases in the patient, shown in Figure 5. While, Figure 6 shows the side effects of breathing pure oxygen at high pressure.

## 7. Components of oxygen concentrators

**Figure 7:**
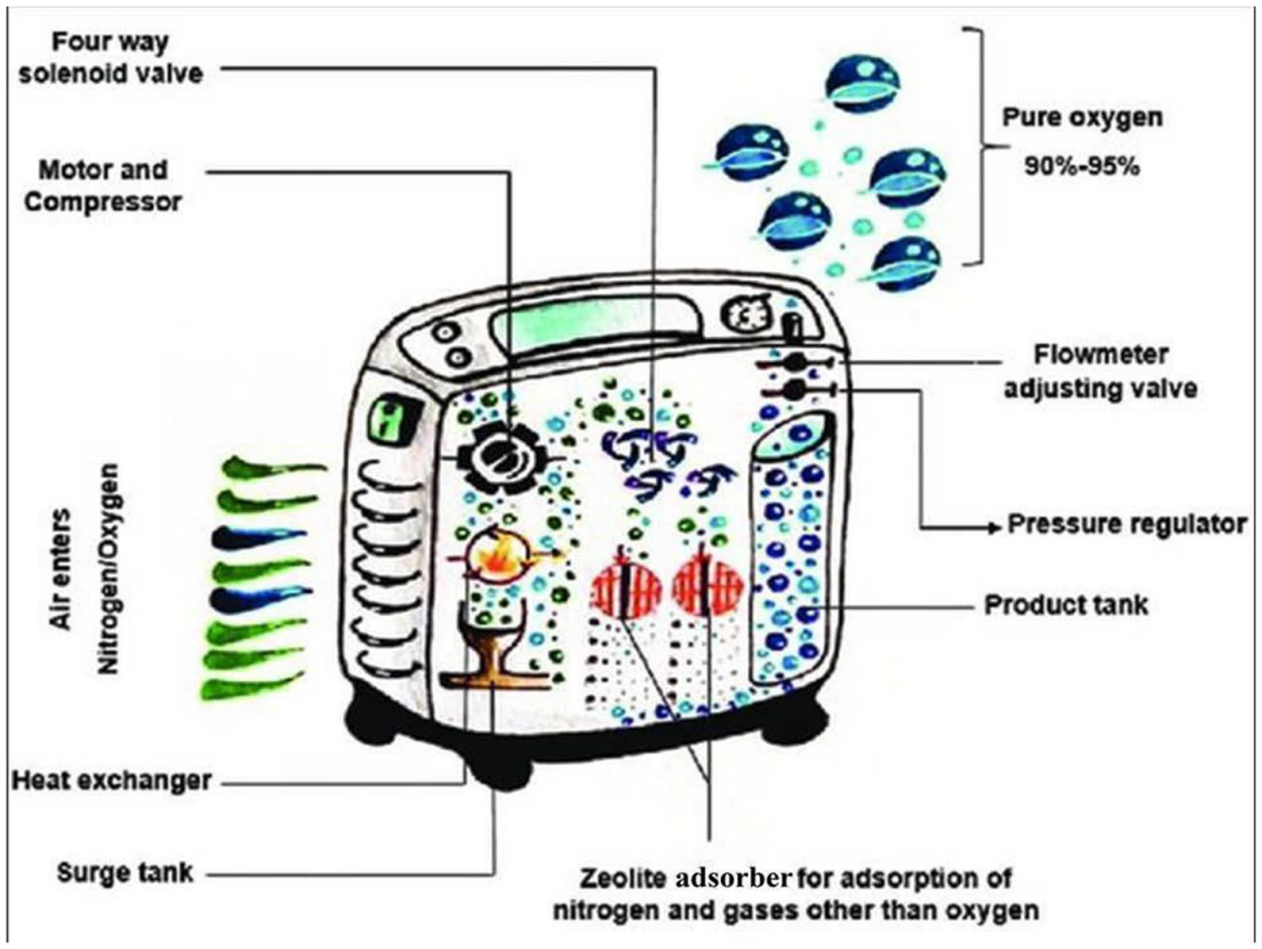
Various components of an oxygen concentrator [adopted from Todur et al. (47)].

The oxygen concentrators have several components like control, filters, air inlet, waste outlet, and flow splitter (47). The control comprises the power switch, oxygen cock, and valve for adjustment below (48, 49). A series of filters are arranged in order, external coarse (gross particle filter), pre-filter, and inlet filter (compressor inlet filter (0.1 μm) in the oxygen concentrators. Besides this, it has a compressor (20 PSI), heat exchanger, 4-way solenoid-controlled valve, sieve canisters with zeolites, pressure equalization valve, check valve, pressure regulator, product canister, outlet filter (0.3 μm), and flow meter (50, 51). The zeolite crystals present in canisters have an average lifespan of 60,000 h (49). Figure 7 shows various components of an oxygen concentrator.

## 8. Principle of oxygen concentrators

**Figure 8:**
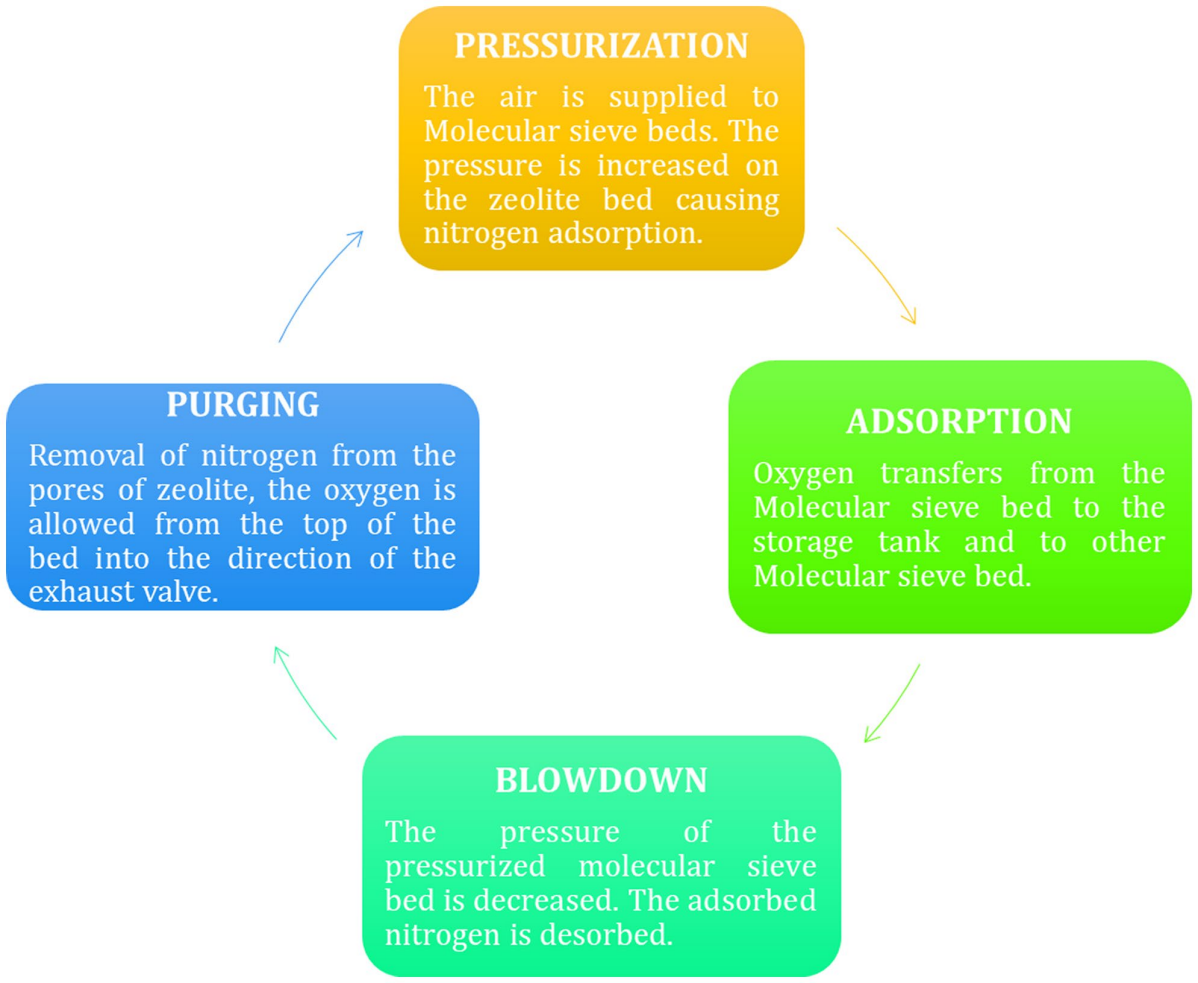
Four stages of PSA cycle (51).

Oxygen concentrators work on the principle of “*pressure swing adsorption*” (PSA) technology (52, 53). The word “*pressure swing adsorption*” implies that the applied pressure keeps on swinging, and consequently, there is continuous adsorption and desorption of gases, i.e., two stages from the molecular sieves. Two stages work in alternation, 1. Adsorption/Production 2. Blowdown/Purge (52). While Figure 8 shows detailed four phases of PSA technology of an oxygen concentrator. The compressed air (21% O_2_+ 78% N_2_) is supplied to one of the columns present in the canister. These columns are filled with adsorbent zeolite materials made of aluminum-silicate hydrates (54).

Firstly, column one gets compressed air, during which N_2_ gets adsorbed on the zeolites’ surface while the O_2_ (present in lower content in air) is passed through these zeolite columns and is collected in an oxygen tank. Due to the continuous adsorption first column gets saturated, so the valve closes, and pressurized air is moved into the second canister containing molecular sieves (55). During this period, the pressure on the earlier column gets lowered, due to which the adsorbed nitrogen gas is released into the surrounding. This particular step, where the column releases the N_2_ from the column and gets prepared for the next cycle, is called regeneration (56). Again, in the next cycle, another column will be regenerated by releasing the adsorbed N_2_, this adsorption and desorption continue due to a swing in pressure; hence it is called “*pressure swing technology*.”

## 9. Working of oxygen concentrators

A stable supply of oxygen-enriched airflow is delivered by oxygen concentrators. Devices called oxygen concentrators (also known as oxygen generators) drive indoor air through several filters to eliminate particles, germs, and other impurities (57). The first stage in the concentration procedure includes pumping air into one of the two cylinders, which comprise a semi-permeable membrane or a “molecular sieve” element. Next, nitrogen is removed, generating pure oxygen (90 percent or more) and a minor amount of certain other gases present in indoor air (58–61). An accessible source of oxygen-enriched air is an oxygen concentrator. Nitrogen is desorbed and pulled outside into the environment simultaneously in another tank. The second step includes the cylinders’ functionality being reverted in a scheduled loop to ensure that patients receive a steady oxygen supply. Certain kinds of high-flow oxygen concentrators can produce up to 10 L/min of oxygen, whereas low-flow oxygen concentrators typically give an oxygen flow of 0.5–5 L/min (62, 63).

In essence, continuous flow dose administration and pulse mode distribution are indeed the two different ways oxygen is administered in oxygen concentrators. While pulse mode delivery pulses an oxygen “bolus” (57) whenever the user starts to breathe, continuous flow dose treatment provides a consistent, smooth, and efficient oxygen flow depending on the configuration value of Lmin^−1^.

**Figure 9:**
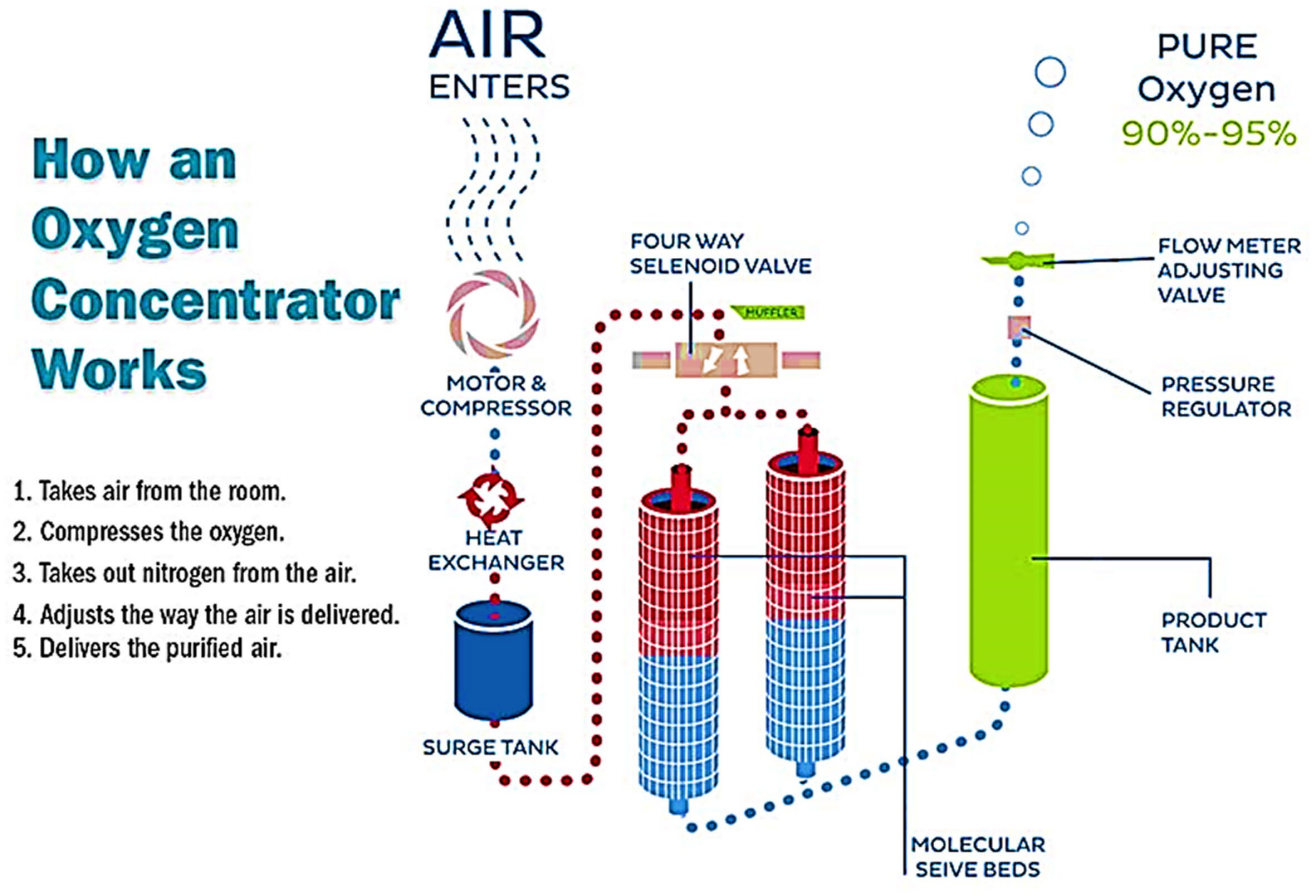
Working of an oxygen concentrator.

Since it provides oxygen through the cannula whenever you breathe, the pulse dose setting is typically utilized during the day. Additionally, pulse dosage concentrators have a much more compact design as well as a better battery life (63). The working principle of an oxygen concentrator is shown below in Figure 9.

## 10. Types of oxygen concentrators

Both stationary and portable oxygen concentrators are available. A continuous oxygen supply is provided by stationary (home) concentrators, which have an average weight of around 10 kg and a flow rate of 0.5 to 10–15 L/min. For long-term oxygen therapy (LTOT) users who want a smaller, portable oxygen supply in a movable apparatus, portable oxygen concentrators are indeed the latest innovation available (64, 65). Weight, size, oxygen flow settings, range of L/min, battery life, and other characteristics of portable concentrators differ (63). Table 2 shows the major differences between a portable oxygen concentrator (POC) and a stationary oxygen concentrator (SOC).

**Table 2:** Major differences between portable and stationary oxygen concentrators.

Portable oxygen concentrator	Stationary oxygen concentrator
Small, lightweight	Large in size, heavy in weight (mean wt ~ 10 kg)
Greater flexibility with power sources	Lesser flexibility with power sources
Lower oxygen output and high cost	higher oxygen output and lower costs

**Figure 10:**
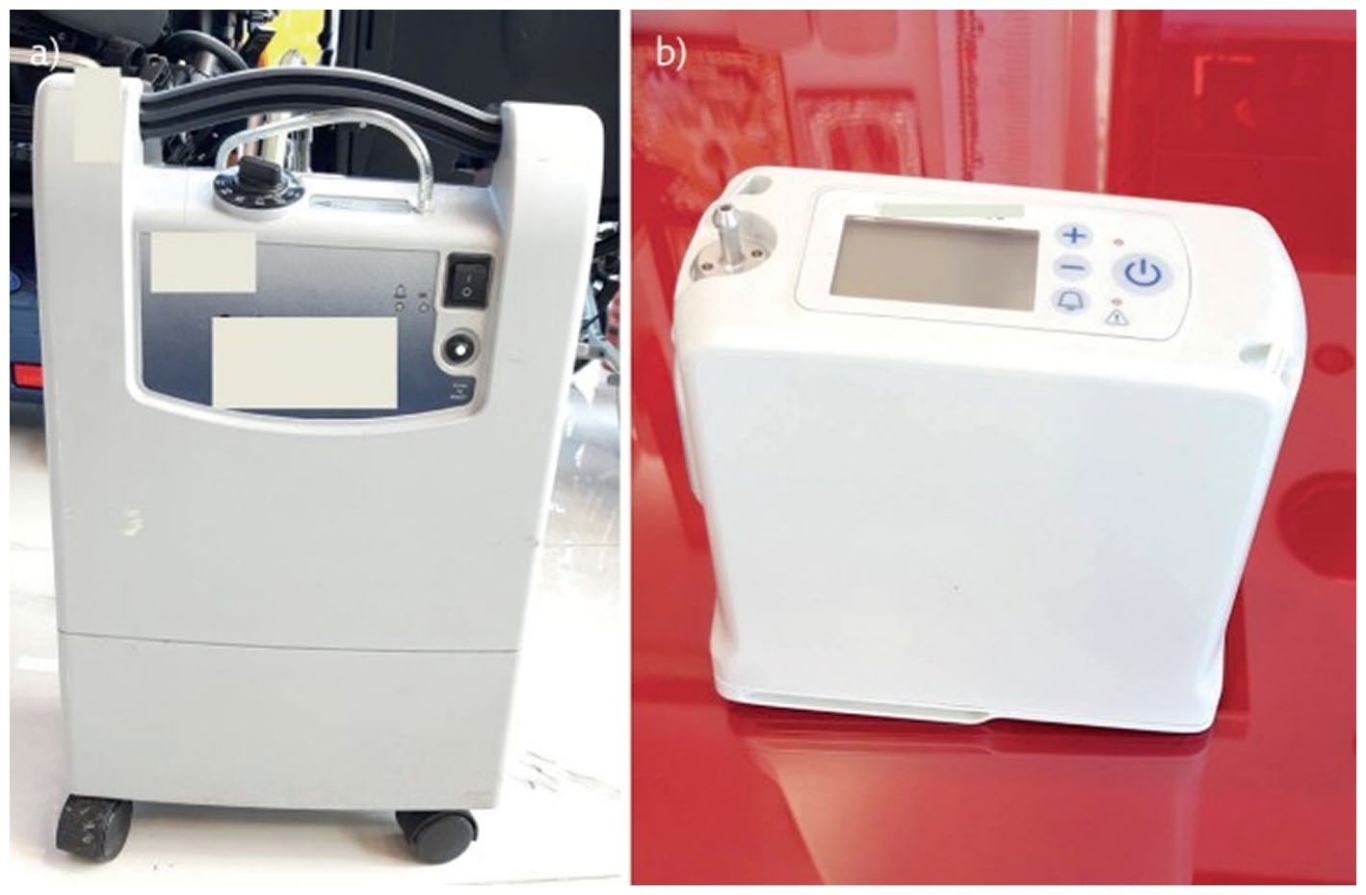
Oxygen concentrators. **(A)** Stationary oxygen concentrator. **(B)** Portable oxygen concentrator adapted from Hardvella (63).

A more recent choice is a super-compact home concentrator, which might weigh as little as 4.5 kg. These portable devices operate on both alternating current (AC; for example, from a wall outlet) and direct current (DC; for example, from a cigarette lighter socket; e.g., they can be smoothly shifted from one place to the other or they can be transported by car during travel). Presently, these can handle oxygen flow rates of up to 2 L/min (63). Zeolites are used in various processes to improve product quality, processing rates, energy efficiency, and the environmental consequences of the oxygen purification process (66). Figure 10A is a stationary oxygen concentrator, while 10B is a POC.

## 11. Clinical applications of oxygen concentrators

**Figure 11:**
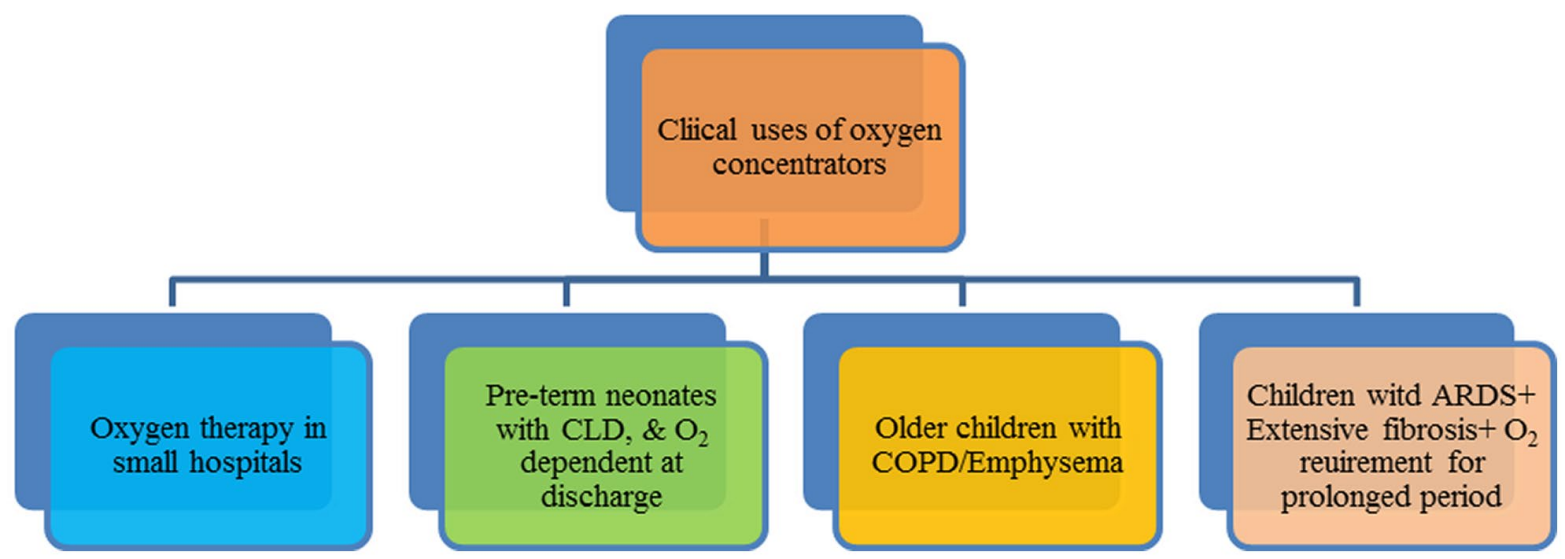
Clinical applications of oxygen concentrators.

In the medical profession, there have been four main instances under which oxygen concentrators are being used: oxygen therapy in small hospitals; oxygen therapy for pre-term neonates with chronic lung disease who need oxygen after discharge; oxygen therapy for older children with chronic obstructive pulmonary disease, pneumonia, pulmonary embolism, or emphysema; and oxygen therapy for children with acute respiratory distress syndrome (ARDS) with extensive fibrosis who need O_2_ therapy for a prolonged period. Besides this, oxygen concentrators could be used in hospitals during surgery to maintain tissue oxygenation during anesthesia and the resuscitation of patients (67). Figure 11 shows the clinical applications of oxygen concentrators.

## 12. Supportive data regarding utilization of oxygen concentrators

Ress and Dudley (1998) (68) reported the role of oxygen therapy, especially oxygen or oxygen concentrators for patients being treated at home. Employing a recently designed polymer of poly [1-(trimethylsilyl)-1-propyne] with an oxygen permeability of 61 × 10 cm^3^ (STP) cm/cm^2^ s cmHg, which itself is 17 times greater than those of the membrane material of traditional concentrators, Akutsu et al. in 1990 built a small, light-weight oxygen concentrator. The oxygen and nitrogen selectivity was 1.80. The dimension and weight of the O_2_ concentrator were about 325 × 180 × 150 mm 4.0 kg, respectively. It generated an oxygen concentration of about 30%, and the maximum flow rate was 41/min (68). Ritz et al. (69) suggested using oxygen concentrators during mass causality situations. A range of oxygen concentrator layouts is accessible, including smaller, more portable units that may serve a single patient and bigger units that could also serve a group of patients or an entire facility (69).

Pollock and Natoli (70) reported the use of chemical oxygen generation for oxygen therapy. To assess the effectiveness of the emOx mobile non-pressurized emergency powdered oxygenation delivery system. This product, which chemically creates oxygen, is promoted to be used as urgent first aid until trained medical support is accessible (70). According to McCoy et al. (71), there are now more possibilities for home oxygen therapy equipment than there were a few years ago due to technological advancements, financial aid from third-party providers, and patient demands. With the development of intermittent-flow delivery, oxygen concentrators that yield 99 percent oxygen authenticity have replaced packaged gas systems (oxygen concentrators) that delivered oxygen that was 99 percent pure, according to the United States Pharmacopeia (71, 72).

In patients with chronic obstructive pulmonary disease (COPD), Yanez et al. (73) documented oxygenation using a single portable pulse-dose oxygen-conserving device and integrated stationery and portable oxygen delivery systems. They said that portable oxygen devices, i.e., oxygen concentrators, simplify and facilitate patient therapy. The subjects chose to use a single portable oxygenation system when in an ambulance or at home. Moreover, fixed and portable systems functioning together generated higher amounts of oxygen than portable systems working alone (73). Katz et al. (74) analyzed the oxygen uptake during rest and exercise in mild COPD patients using portable oxygen concentrators in order to improve the effectiveness of oxygen generation and nitrogen adsorption in oxygen concentrators (74).

Pan et al. (75) suggested the usage of nanosized zeolites. They suggested the device as a basic requirement for lung infection-associated patients. To effectively adsorb nitrogen from the air, a 13X nanosize zeolite with Li^+^ exchanged is utilized as the adsorbent. To better understand the pressurization and depressurization processes taking place inside the microporous region of nanosized zeolites, a dynamic model of the pressure and vacuum swing adsorption units was created (21). The effectiveness of continuous versus pulsed oxygen administration was compared by Chen et al. in 2017 utilizing an accurate adult nasal airway replica. The main objectives of this work were to examine steady flow (SF) from a compressed oxygen tank to PF for a commercial POC and to construct a prospective *in vitro* analysis for inhaling oxygen supply employing a set of plausible airways replicas (75).

In a pediatric ward in Malawi, Meyers et al. in 2018 documented employing bubble continuous positive airway pressure (bCPAP) to treat seriously unwell children. Humidified bCPAP air/oxygen flow was given by customized oxygen concentrators or oxygen cylinders. Further research is required to determine the function of bCPAP and other non-invasive ventilator support methods in an effective healthcare program for seriously sick children with MOF at the tertiary and district hospital levels in low-resource economies (76). Branson in 2018 stated that patients with COPD and severe resting hypoxemia were shown to have a greater chance of survival when receiving long-term oxygen treatment (LTOT) at residence. It has already been demonstrated that oxygen supplementation administered by an oxygen concentrator during physical activity and exercise reduces symptoms and maintains arterial oxygen saturation, but it does not enhance long-term effectiveness (77). Melani et al. (78) reported the importance of oxygen-producing devices (O_2_ concentrators) in home oxygen therapy. Besides this, they also explained the role of a pulse oximeter, etc., in-home oxygen therapy (78).

Pulsed oxygen delivery through portable oxygen concentrators against continuous flow oxygen delivery was compared in an *in-vitro*-*in silico* research by Chen et al. in 2019. The effectiveness of four POCs with continuous flow oxygen was compared using *in vitro* bench measurements and *in silico* numerical simulations by anticipating the FIO2 at the trachea and entering the acini. In respect of volume-averaged FIO2, continuous flow oxygen provided adequate (>2% absolute) oxygen for all nominally equivalent pulse flow levels of >2 (79). Bench-wise comparative research between modern portable oxygen concentrators and various breathing techniques was conducted by Martin et al. in 2019. Considering three realistic breathing patterns, resting, and oronasal inhalation while sleeping, three modern devices were bench-tested (80). In Madrid, Spain, patients undergoing portable oxygen therapy were the subject of a telephone-based study conducted by Alises et al. in 2019. The survey looked at patients’ present situations, behaviors, and perceptions.

Patients selected from a registry undergoing treatment with portable oxygen were interviewed over the phone. Most patients used mobile oxygen concentrators (99.59 percent) (81). According to Litch and Bishop (82), using oxygen concentrators to give medical oxygen in isolated, high-altitude environments significantly impacts supply and related costs. In a rural hospital located at 3,900 m in the Nepal Himalayas, researchers highlighted the significance of oxygen concentrators. The investigators also stated that oxygen concentrations of more than 80% might be sustained at delivery flow rates of 2–5 L per minute (82). According to the findings of the research conducted by Sardesai et al. (83), portable oxygen concentrators are recommended to be utilized for home-isolated COVID patients. This is particularly the case for patients who do not exhibit severe symptoms and for whom there is no requirement for BIPAP. Through the follow-up with COVID-19 patients undergoing home oxygen therapy, feedback was obtained (83).

According to Shen in 2020, oxygen therapy has the potential to improve the antiviral response of the immune system. Treatment with nighttime oxygen may stop the progression of COVID-19. SARS-CoV2 is known to use ACE2, which is the same cell invasion receptor as SARS-CoV2. It is secure and simple to apply in therapeutic settings (a home O_2_ concentrator is enough for a patient) (84). According to Duke et al. in 2020, oxygen therapy is a crucial medical treatment and a critical part of functional hospital setups. They emphasized the application of oxygen concentrators in especially low-income countries, where medical facilities are not so well developed. So, it is best suited for African and Asia Pacific countries, which lack better medical infrastructure (85).

Cuerpo et al. (86) suggested the use of oxygen concentrators along with the noninvasive ventilation device in interstitial lung disease patients, which can improve home oxygen therapy. They employed both oxygen concentrators alone and in combination with non-invasive ventilation, and they discovered that the average SaO_2_ increased to 91% (88–93) versus 88% (86–90%); *p* = 0.0005 as well as a decline in the measure of time with oxygen saturation below 90%: 36% (6–56%) versus 58% (36–77%); *p* 0.0001 (86). In Sabah, Malaysia, during the COVID-19 pandemic, Cheah et al. (87), recommended using a dual oxygen concentrator system for manual breathing. According to the investigators, oxygen concentrators are a life-saving alternative for patients, and they could even save a severely ill patient during COVID (87). For use in medical applications, Vemula and Matthew in 2021 designed an experimental “Snap-on” and an independent single-bed oxygen concentrator. For continuous oxygen supply, a unique single-bed, “Snap-on,” and independent medical oxygen concentrator design based on a rapid pressure swing adsorption technique was explored. When connected to a preexisting compressed gas stream, the Snap-on concentrator’s configuration makes it simple to use the devices and efficiently produce oxygen for medical uses. Because of its separate compressor and ease of portability, the Snap-on concentrator is extremely significant for oxygen therapy, meeting the demands of a higher number of patients in the circumstances like COVID-19 (56).

In 38 remote health institutions in nine regions of Papua New Guinea, Pulsan et al. (88) examined a scheme for enhancing credible oxygen therapy using oxygen concentrators, pulse oximeters, and renewable solar power. In rural and remote hospitals and healthcare institutions, solar-powered oxygen systems can be installed on a broad scale and have been linked to a decrease in child fatalities and a reduction in referrals (88). According to Madaan et al. (89), rural India’s oxygen needs should focus on employing a supply of substantially pure oxygen that is secure, affordable, simple to access, and mass-producible. The arrangement of a self-sustaining oxygen concentrator (pressure swing adsorption with multiple molecular sieve technology) that provide oxygen at high flow rates through a centrally controlled distribution network to 100 or even more bedded community hospitals, with backup from an oxygen bank of 10 × 10 cylinders, may be able to achieve this. It could also act as a facility for replenishing local oxygen cylinders for medical emergencies inside the hospital premises and for delivery to ambulances, primary health centers, and other remote locations (89).

## 13. Types of zeolites used in oxygen concentrator

Zeolites are micron-sized, crystalline, porous compounds made up of alumina and silica hydrates along with an alkali metal (90). Its structure has numerous pores, making it suitable as an adsorbent. It is widely used in the oxygen concentrator due to its ability to withstand higher temperatures and its tendency to regenerate completely aster adsorption (51). Zeolite molecular sieves for oxygen concentrators come in two primary varieties: sodium type and lithium type (91). The oxygen concentrator is compact and easy to transport because lithium-type zeolite molecular sieves are much more effective than sodium-type zeolite molecular sieves that decreases the amounts of oxygen generation. Since sodium oxygen molecular sieve is considerably less expensive than lithium molecular sieve, sodium oxygen molecular sieve is also readily available in markets.

All the above factors make oxygen concentrators more economical and environmentally friendly (51). Recently Sami et al. (51) studied various types of zeolites used in oxygen concentrators and concluded that there are mainly four types of zeolites used as adsorbents. These four types of zeolites are 5A zeolite, Oxysiv 5, 7 & Sylobead MS S 624, LiX zeolite, and LiLSX zeolite. As per their investigation, it was concluded that various absorbents in oxygen concentrators have different cycles and purity of oxygen produced by them (51). The properties of these four commonly used zeolites are summarized in Table 3.

**Table 3:** Literature survey on zeolites used for oxygen concentrators adopted from Pan et al. in 2017 and Semi et al. in 2022 (92).

Absorbent	Cycle type	Cycle duration(s)	Operating pressure (bar)	Purity (%)	Flow rate
5A zeolite	PSA 2-step	100	Ph = 1.52, P1 = 1.01	85	–
Oxysiv 5,7 & Sylobead MS 624	PSA 2-step	18	Ph = 3, P1 = 1.945	94.5	3.7
LiLSX zeolite	PSA 2-step	3–9	Ph = 4, P1 = 1	90	1–3
Lix zeolite	PSA 2-step	3–5	Ph = 3.04–4.05, P1 = 1.01	90	5

## 14. Drawbacks of micron-sized molecular sieves

Since the size of the currently used molecular sieves is in microns, less nitrogen could be adsorbed on their surface due to size. Consequently, the efficiency of oxygen generation, as well as the purity of oxygen, are both less. So, to overcome this, ultrafine zeolites have to be used to enhance the efficiency and purity of the oxygen. Zeolites are widely used in oxygen concentrators to adsorb the nitrogen on their surface. This process takes place under pressure swing adsorption technology, which concentrates oxygen from the air. The zeolites have more affinity for nitrogen and less affinity for oxygen. Since most oxygen concentrators use micron-sized molecular sieves to adsorb the nitrogen, their efficiency is not 100%; rather, it falls to 87–93%, depending on several other factors (92).

## 15. Role of nanotechnology in oxygen concentrators

There are several procedures by which the efficiency of such oxygen concentrators and the purity of oxygen can be achieved. One such process is the application of nanozeolites, which are used for this purpose since they are smaller in size and contain more reactive sites on their surface (93). Nanozeolites are a type of zeolites which are produced from the molecular zeolites and these have particle distribution and sizes of <200 nm. Due to their small size, these nanozeolites exhibit significant surface–to–volume ratios (SVR) and large surface values. More nitrogen will be adsorbed on the surface of nanozeolites compared to micron-sized zeolites because of the high surface area-to-volume ratio (94–96).

Some investigators have used nanozeolites in oxygen concentrators for nitrogen adsorption and obtained satisfactory results. Some of the examples are provided below in chronological order. In a continuous adsorption and desorption cycle with a cycle period of 90 s, Pan et al. (92), employed nanozeolites as molecular sieves in which the outcome of the oxygen concentrator is a flow of enriched oxygen at about 90 vol percent. The adsorption column measures 20 cm in length and 3 cm in diameter. To effectively adsorb nitrogen from the air, a 13X nanosize zeolite with Li^+^ exchanged is utilized as the adsorbent. To better understand the pressurization and depressurization processes taking place inside the microporous region of nanosized zeolites, a simulation of the pressure and vacuum swing adsorption units was devised (92). Besides this, several investigators have also reported the surface modification of such nanozeolites by other metallic particles to make the process more efficient (1, 97–99).

In this regard, chabazite (CHA) zeolites were manufactured at different range diameters of 120–300 nm, whereas the smaller sample (120 nm) also consisted of tiny particles (around 20 nm) (100). These small units of the CHA zeolites are basically used along with zeolites for gas separation technologies. The design and development of such nano-sized zeolite-based specimens are highly appreciated for constructing vacuum swing adsorption devices due to their thermal stability and compressive stress. Moreover, the hybrid CHA zeolites are prepared by doping a few desired ions, such as Sr^2+^, K^+^, and Na^+^ cations, by avoiding organic substances (100). The as-prepared membranes are useful to form a chemically stable system as the cation species interact well with the negatively occurring aluminum oxygen sites (AlO_2_)^−^ in a tetrahedral geometry (101). Nowadays, various models have been explored to design and develop zeolite-based oxygen concentrators. For example, a molecular sieve with particular model numbers and activated alumina in PSA oxygen generators.

The higher production of O_2_ includes a larger amount of nitrogen adsorption. Additionally, the selectivity in N_2_ and O_2_ (nitrogen/oxygen) allows it to be called with different model names, such as the JLOX-500 series molecular sieve. Using nanotechnologies, these systems show better wear resistance and good service rate as the pore sizes, surface, and structural modifications are carried out under preparation procedures (102). The use of nano-based techniques felicitates a cost-effective oxygen production with lower energy consumption by using the nano-sized zeolites and their derivatives using metal cations and organic units. Thus, the chemical structure of the zeolite is extensively recommended for the efficient oxygen concentrator due to its chemical and physical strength, including its (SiO_4_)^4+^ and (AlO_4_)^5+^ tetrahedral geometry sharing the partially negatively charged oxygen atoms. Extensively, the isotherm concept has been derived from understanding the isotherm adsorption of the zeolites using Langmuir, Freundlich, and Tempkin model’s theorem, and the characteristics of adsorption application are being studied (103).

Pseudo-first order, pseudo-second order, Bangham, intra-particle diffusion, and Elovich models were used to assess the kinetics of the adsorption objects, demonstrating the feasibility of the applicability of gaseous adsorption (103). As oxygen concentrators basically include gas compressors and adsorption sciences, nano-sized zeolites have been encouraged in this sector. Overall, stronger structural aspects with the change in cationic oxidation states help to modify the framework of the zeolite, which affects the adsorption nature and separates the gases, mainly O_2_ and N_2_/O_2_. These physicochemical properties of the synthetic zeolite with their morphological changes could be widely used in modern nanotechnology and sciences toward newly designed oxygen concentrators/systems and their accessories (104–106). Overal evolution in use of molecular zeolites to nano-zeolites has gained importance to tackle the O_2_ supply chain issue that had been severely felt in medical care during the pandemics in developing countries such as India and similar countries (107–110).

## 16. Conclusion

Oxygen is one of the basic and essential medical drugs required by patients in less to highly severe cases. The demand and supply of mobile oxygen generators have increased drastically in the last couple of years. So, portable oxygen concentrators, initially proven life-supportive in hilly areas in African countries, have now gained importance. Its economical, mobile natures, and ease of handling, have made basic needs in every house. The utilization of four basic types of zeolites as an adsorbent has limitations in the sense of generation, purity of oxygen, etc., and efficiency being micron in size. So, the nanosized zeolites can potentially make the process of adsorption and desorption in oxygen concentrators more effective due to their small size and high surface area-to-volume ratio. Nanosized zeolites have the potential to be a game-changer in the delivery of pure oxygen to the patient in the face of current and future pandemics caused by a wide range of viruses and bacteria.

## Author contributions

VY, BP, and DKS: conceptualization, data curation, formal analysis, funding acquisition, investigation, methodology, project administration, resources, software, supervision, validation, visualization, roles and writing – original draft, and writing – review and editing. NC, GI, AR, BiS, and BhS: concept, writing – original draft, writing – review and editing, and revision. All authors contributed to the article and approved the submitted version.

## Conflict of interest

The authors declare that the research was conducted in the absence of any commercial or financial relationships that could be construed as a potential conflict of interest.

## Publisher’s note

All claims expressed in this article are solely those of the authors and do not necessarily represent those of their affiliated organizations, or those of the publisher, the editors and the reviewers. Any product that may be evaluated in this article, or claim that may be made by its manufacturer, is not guaranteed or endorsed by the publisher.
